# Evaluation of nutritional behaviour related to COVID-19

**DOI:** 10.1017/S1368980020004140

**Published:** 2020-10-19

**Authors:** Funda Elmacıoğlu, Elif Emiroğlu, Mutlu Tuçe Ülker, Berkin Özyılmaz Kırcali, Sena Oruç

**Affiliations:** Department of Nutrition and Dietetics, Faculty of Health Sciences, Istinye University, Topkapı Campus, Maltepe Neighbourhood, Teyyareci Sami Street, Building no.3, Zeytinburnu, İstanbul 34010, Turkey

**Keywords:** Coronavirus infection, Nutritional behaviour, Nutritional sciences

## Abstract

**Objective:**

It is known that social isolation process has an impact on individuals’ eating behaviours. Continuing nutritional behaviour resulting from emotional eating, uncontrolled eating and cognitive restriction may turn into eating disorders in the future. The purpose of this study is to evaluate the possible effects of Corona Virus Disease-2019 (COVID-19) pandemic and social isolation process on individuals’ nutritional behaviours and body weight changes.

**Design:**

Retrospective cohort study.

**Setting:**

Nutritional behaviours of the participants before the COVID-19 pandemic and in the social isolation process were evaluated with the Three Factor Nutrition Questionnaire. The changes in individuals’ body weight during this period were also evaluated.

**Participants:**

A total of 1036 volunteer individuals (827 women, 209 men) aged 18 years and over participated in the study.

**Results:**

During the COVID-19 pandemic and social isolation process, there was an increase in emotional eating and uncontrolled eating behaviours of individuals, but no significant change in cognitive restriction behaviour occurred (*P* = <0·00; *P* = <0·00 and *P* = 0·53, respectively). It was reported that the body weight of 35 % of the individuals who participated in the study increased during this period.

**Conclusion:**

Social isolation process practiced as a result of COVID-19 pandemic may lead to changes in some nutritional behaviours. Some precautions should be taken to prevent this situation that occurs in nutritional behaviours from causing negative health problems in the future.

An epidemic of pneumonia, which is thought to be caused by a new coronavirus, occurred in late 2019 in the city of Wuhan, China, and this epidemic was not controlled and spread worldwide^(1)^. The virus held responsible for this process has been named as ‘new coronavirus-2019’ (2019-nCoV) and is named as ‘Severe Acute Respiratory Syndrome-Coronavirus-2’ (SARS-CoV-2) by the WHO. The disease which is caused by the virus has been described as Corona Virus Disease-2019 (COVID-19)^(2,3)^. Infectious diseases caused by zoonotic coronaviruses such as SARS-CoV-2 have created a global public health concern. It was declared as a pandemic and emergency by WHO^(4)^.

From the moment of the emergence of the virus, WHO has issued social isolation warnings and published some reports on the importance of isolation for SARS-CoV-2, which has a high infectious rate and mortality rates as it finds new hosts every day^(5,6)^. Due to the increase in the incidence of COVID-19 cases despite rigorous international control and quarantine efforts, guidelines with the recommendations of the importance of individual hygiene (especially hand hygiene), food hygiene and safety, nutritional and physical activity to support the immune system have been prepared by the international authorities to facilitate the fight against the pandemic^(7,8)^.

It is known that the social isolation process announced due to pandemic has a direct effect on many parameters including individuals’ eating behaviours^(9)^. Eating behaviour is the tendency caused by all the knowledge, thoughts, feelings and behaviours of individuals related to nutrition. Social, cultural and demographic factors, physiological characteristics of the individual, past experiences with food and geographical factors are effective in shaping the eating behaviour. Excesses in each of these subcategories cause eating behaviour problems^(10,11)^.

Uncontrolled eating behaviour is consuming excessive amounts of food that individuals do not need by losing their control. People who have uncontrolled eating behaviour tend to consume excessive amounts of food and to have a tendency to be addicted of certain types of foods. Overeating and obesity have similar results such as getting more energy than needed^(12)^.

Different eating actions of individuals according to their emotional states are expressed as emotional eating, and they are defined as the tendency to eat to deal with negative emotions such as depressed mood, anger, anxiety and worry. Emotional eating is basically based on two assumptions. The first one is that the negative emotions encourage the individual to eat, and the second one is that the action of eating reduces the intensity of the negative emotions^(13)^. Studies examining the relationship between different emotional states and food intake indicate that eating tendencies of individuals increase in times of boredom, depression and weakness, while eating tendencies and food intake decrease during fear, tension and pain. While individuals prefer healthy foods in positive emotional states, they tend to consume more junk food in negative states^(10,13)^. Among the eating behaviours that are thought to be related to each other, it is stated that emotional eating behaviour causes more food intake than normal amounts in negative emotional states such as loneliness, depression and anger; uncontrolled eating behaviour causes uncontrolled food intake tendency – which is much more than normal amounts, overeating –cognitive restriction behaviour causes deliberate restriction of food intake in order to achieve weight loss or control body weight. It is estimated that cognitive restriction behaviour causes eating disorders such as binge eating syndrome^(14,15)^.

Eating behaviour has a crucial role in energy balance and weight control. Changes in the eating behaviour may have an effect on losing and gaining weight^(16)^.

This study was planned to evaluate the effects of COVID-19 pandemic process on individuals’ nutritional behaviours and body weight changes. With the findings to be obtained from the study, it is aimed to identify nutritional problems that may occur in the process after the pandemic and take precautions by evaluating the nutritional behaviours of the society during the pandemic.

## Material and methods

### Research model, population and sample

This study is an online research to evaluate the possible changes in the nutritional behaviour of individuals during the social isolation process. Within the scope of the study, 1050 volunteers from sixty-four different provinces of Turkey were reached in May 2020. A total of fourteen participants were excluded from the study because they were under the age of 18 years. Thus, the sample of the research consisted of 1036 people.

The study was conducted in accordance with the Helsinki Declaration. Participants were informed about the study, and their consent was obtained through *Google Forms*.

During the presentation of the online survey to the participants, the privacy conditions imposed by the Google Forms regarding the determination of the storage period and not sharing the data with third parties were complied with.

### Data collection

The first case of COVİD-19 in Turkey was seen on 10 March 2020. Social isolation had started on 17 March 2020. Data collection of this study was started on 6 May and ended on 26 May and lasted for 3 weeks. Nutritional behaviour and body weight changes during the process were analysed and examined in this study. Participants were advised shelter in place, be more careful at getting secure food, providing hygiene, shopping online and social distance. Also, curfew at weekends and national holidays was implemented till June. People who are older than 65 years, younger than 20 years and have chronic disease are banned to go outside except certain hours in a week. During the process of the curfew, people are not restricted about getting food, and people are advised not to stock because they can do shopping most of the time by paying attention to the requirements to be protected from the virus.

In the study, individuals were reached through an online questionnaire created using *Google Forms*. The survey includes questions on anthropometric measurements, such as body weight and height, in addition to socio-demographic information such as age, gender, educational background and city of residence. The revised Three Factor Nutrition Questionnaire (TFEQ-R18) has been used to assess the nutritional behaviour of individuals in the COVID-19 pandemic and social isolation process. The questions in the scale were asked retrospectively, covering the pre-social isolation process, and the changes in the nutritional behaviour of individuals were interpreted by analysing the difference between these values. TFEQ-R18, developed by Stunkard and Messick and revised by Karlsson *et al.*, has been translated into Turkish by Kıraç *et al.* and its validity and reliability have been proven. With this questionnaire, it is possible to measure the degree of deliberate eating restrictions, uncontrolled eating and emotional eating^(17,18)^.

The scale examining uncontrolled eating, cognitive restriction and emotional eating behaviour consists of eighteen items in total. It has a four-point Likert-type rating (1 is absolutely true; 2 is mostly true; 3 is mostly false and 4 is absolutely false). The scoring has been done as follows by taking the studies carried out to standardise the distribution of unequal questions as examples:
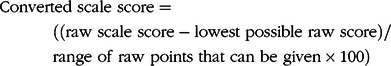



The converted scale score ranges from 0 to 100, and the high score is considered as an indication that cognitive restriction, emotional eating and uncontrolled eating behaviour are stronger in individuals^(17,18)^.

The change in the body weight of individuals during this period was determined according to their own declaration. Individuals were evaluated in four categories: body weight increased, body weight decreased, body weight did not change and unknown. The number and percentage values for these data were obtained.

In the questionnaire, the participants were asked about their current body weight and height values, and the individuals’ BMI values were calculated by the researchers according to the formula BMI = body weight/height length^2^, and individuals were classified as underweight (below 18·5 kg/m^2^), normal weight (18·5–24·9 kg/m^2^), pre-obesity (25·0–29·9 kg/m^2^), obesity class I (30–34·9 kg/m^2^), obesity class II (35·0–39·9 kg/m^2^) and obesity class III (above 40 kg/m^2^) according to WHO criteria^(19)^.

### Statistical evaluation

The data obtained from the survey were analysed using the SPSS 22.0 package program (IBM SPSS Statistics for Windows, Version 22.0). For continuous variables, arithmetic mean ± sd; for categorical variables, frequency distribution and percentage values are given. Whether the distribution of the data was normal was evaluated with the Shapiro–Wilk test; and TFEQ-R18 converted scale scores were found not to show normal distribution. For this reason, data on the nutritional behaviours of individuals in the period before social isolation and in the social isolation process were compared with Wilcoxon paired two sample test. Whether the scale scores differed by gender was assessed by the Mann–Whitney *U* Test, and whether they differed by educational status was assessed by the Kruskal–Wallis test. Data with a *P* value below <0·05 were considered significant^(20)^.

An open-ended question that asked the most obvious change in their diet during the social isolation process was proposed to the participants, and this question was analysed by descriptive analysis method. During the analysis, two different researchers divided the responses into eight different codes and sub-themes corresponding to these codes in order to categorise meaningful sections from the answers given to the question. These data are expressed in frequency and percentage^(21)^. During the determination of the codes and themes, the opinions of a different third researcher were taken into consideration in the resolution of disputes between the researchers^(22)^.

## Results

In this research, which aims to evaluate the effects of COVID-19 pandemic and social isolation process on individuals’ nutritional behaviours, data related to the change in nutritional behaviours (uncontrolled eating, cognitive restriction and emotional eating) were collected and evaluated, in addition to the socio-demographic characteristics of the participants such as age, gender and educational status and anthropometric measures such as body weightand height.

In this study, in which 1036 individuals (827 women, 209 men) from sixty-four different provinces across Turkey participated, the average age of the individuals was 33·05 (sd 12·98). Information on some socio-demographic characteristics and anthropometric measurements of the participants are given in Table 1.

**Table 1:** Some socio-demographic characteristics and anthropometric measurements of the participants (*n* 1036)

Characteristics	*n*	%
Age (year)		
Mean	33·05	
sd	12·98	
Gender, *n* (%)		
Female	827	79·80
Male	209	20·20
Education status		
Primary school graduate	16	1·50
Middle school graduate	21	2·00
High school graduate	224	21·60
Associate/undergraduate degree	592	57·10
Graduate degree	143	13·80
Ph.D. degree	40	3·90
City of Residence		
Istanbul	661	63·80
Ankara	47	4·50
Izmir	38	3·70
Other	290	28·00
Body weight (kg)		
Mean	66·99	
sd	15·13	
Min–max	40·00–148·00	
Height (cm)		
Mean	166·91	
sd	9·80	
Min–max	148·00–202·00	
BMI (kg/m^2^)		
Mean	23·98	
sd	4·44	
Min–max	14·88–48·89	

**Fig. 1:**
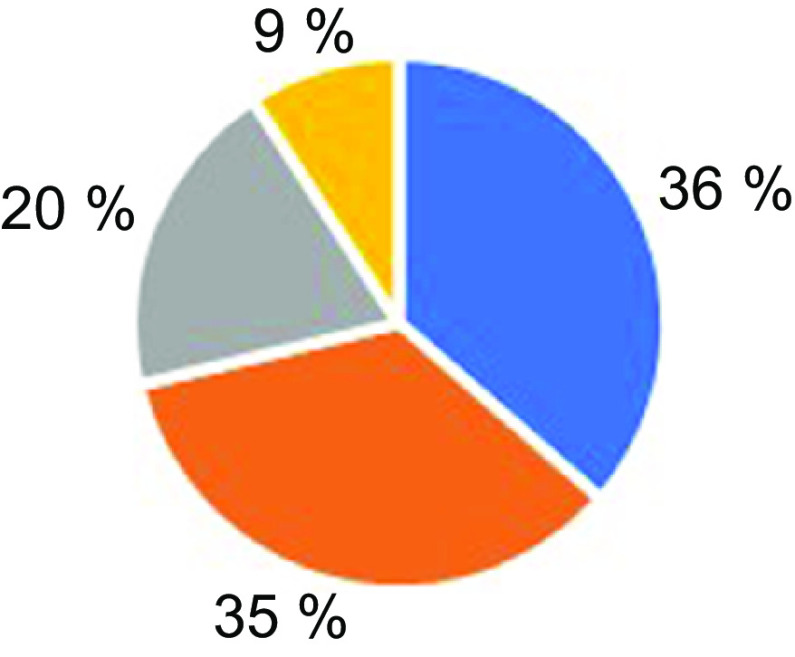
Changes in individuals’ body weight in COVID-19 pandemic and social isolation process (*n* 1036). 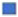, No change occurred; 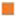, body weight increased; 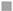, body weight decreased; 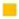, unknown

It was questioned whether a change in body weight of individuals occurred during the COVID-19 pandemic and social isolation process. The results obtained based on the participants’ own declaration show that 35 % of the individuals have increased body weight since the isolation began (Fig. 1).

Nutritional behaviours of individuals in the period before social isolation and in the social isolation process were evaluated with TFEQ-R18. Between these two periods, it was analysed whether there is a difference in individuals’ uncontrolled eating, cognitive restriction and emotional eating behaviours. The study results show that the COVID-19 pandemic and social isolation process lead to an increase in individuals’ emotional eating and uncontrolled eating behaviour; however, it does not make a significant difference in cognitive restriction behaviour. Findings related to changes in individuals’ nutritional behaviour are given in Table 2.

**Table 2:** Changes in individuals’ cognitive restriction, emotional eating and uncontrolled eating behaviours (*n* 1036)

	*n*	Mean rank	Sum of ranks	*Z*	*P* ††
CR after–CR before					
Negative row	329	351·98*	115 802·00	−0·61	0·53
Positive row	342	320·63†	109 654·00		
Equal	365	–‡	–		
EE after–EE before					
Negative row	188	239·81*	45 085·00	−4·94	<0·00**
Positive row	304	250·63†	76 193·00		
Equal	544	–‡	–		
UE after–UE before					
Negative row	328	404·76*	132 762·50	−7·11	<0·00**
Positive row	530	444·81†	235 748·50		
Equal	177	–‡	–		

EE, emotional eating; CR, cognitive restriction; UE, uncontrolled eating.

†† Wilcoxon paired two sample test.

* After < Before.

† After > Before.

‡ After = Before.

** *P* < 0·05.

Possible changes in cognitive restriction, emotional eating and uncontrolled eating scores of individuals according to their BMI classes were analysed with Wilcoxon paired two sample test. The results show that uncontrolled eating behaviour increased significantly in normal and overweight individuals (*Z* = −5·88, *P* = 0·00; *Z* = −3·66, *P* = 0·00, respectively). They also show that emotional eating behaviour increased significantly in normal individuals (*Z* = −3·50, *P* = 0·00).

In the process of social isolation, it was observed that women’s uncontrolled eating and emotional eating scores were higher than men, and their cognitive restriction scores were lower than men, but the differences between the genders are not significant (*P* = 0·48; *P* = 0·12; *P* = 0·06, respectively).

In the process of social isolation, Kruskal–Wallis *H* test was performed to determine whether individuals’ uncontrolled eating, emotional eating and cognitive restriction scores differ significantly according to their educational status, and the difference between their educational status was not statistically significant (*P* = 0·96; *P* = 0·86; *P* = 0·12, respectively).

In the social isolation process, the most obvious change in individuals’ diet was asked to the participants with an open-ended question, and a descriptive analysis of the responses was made. According to the results of the analysis, the answers were classified under eight categories and sub-themes were created. One hundred and fifty one of 899 individuals who answered this question declared that there was no change in their diet. It was determined that the most frequent change was the increase of uncontrolled eating behaviour. Data on other changes in the participants’ diet during the social isolation process are presented in Table 3.

**Table 3:** The most obvious changes in the diet of the participants during the social isolation process (*n* 899)

The most obvious changes in the diet	Frequency (%)	
Increased uncontrolled eating behaviour Increase in portion size Increase in food consumption between meals Not feeling full Eating despite being full	199	22·14 %
No change in the diet	151	16·80 %
Increased consumption of certain types of food Increased consumption of snack foods Increased food consumption with high sugar content Increase in bread and pastry consumption	145	16·12 %
Developing healthy eating behaviour The consumption of foods considered to be healthier (especially foods rich in vitamin C and water)	136	15·13 %
Change of the diet Increase in the number of the main course/snacks Decrease in the number of the main course/snacks Change in mealtimes	124	13·79 %
Developing restrictive nutritional behaviour Behaviour restricting total energy intake Behaviour restricting the consumption of certain types of food	82	9·12 %
Change in ready meals/home food consumption Decreased ordering of food from outside Increased cooking at home Trying different recipes	51	5·67 %
Starting to consume foods that were not consumed before	11	1·22 %

## Discussion

The COVID-19 pandemic causes deaths at a global level and the number of infected individuals increases day by day, leading the governments to decide on quarantine, social distance and isolation. All these measures taken to protect public health by controlling the spread of the virus, and the psychological impact of this process on individuals has caused widespread concerns about overeating, sedentary life and vulnerability to weight gain in most of the society. It is imperative to carry out global actions that support healthy diet and physical activity, both in the process of social isolation and in the post-social isolation period, to encourage people to lead a healthier lifestyle^(23,24)^. This study, conducted with a total of 1036 participants from sixty-four different provinces across Turkey, provided the first data on the orientation and level of nutritional behaviour changes caused by the social isolation practice in the Turkish society and will guide the experts on the health problems and precautions to be taken in the future.

The changes in the nutritional behaviours (emotional eating, uncontrolled eating and cognitive restriction) of the individuals participating in the study were determined with TFEQ-R18. The results show that the COVID-19 pandemic and social isolation process led to a significant increase in individuals’ emotional eating and uncontrolled eating behaviour. Previous research on emotional eating behaviour supports these results. The results of a study conducted by Koball *et al.* in 2012 show that participants tend to eat more in response to depression. In addition to this, the results of this study show that when they feel bored, they eat more often than other emotional changes such as anger, anxiety and depression^(25)^.

In the study, it was also examined whether the nutritional behaviours of individuals differed according to gender in the process of social isolation, and it was concluded that women’s uncontrolled eating and emotional eating scores were higher than men, but this difference was not statistically significant.

In the social isolation process, people are bored with the interruption of the work routine, which is associated with an increase in energy, fat, carbohydrate and protein consumption. Continuous exposure to news about pandemic may further increase stress. This stress causes people to overeat, and the foods that are preferred to consume are generally the foods that make people feel better. The desire to consume a particular type of food is called ‘food craving’ – desire to eat – a multidimensional concept that includes emotional, behavioural, cognitive and physiological processes^(26)^. In this study, the most obvious change in the diet of the participants during the COVID-19 pandemic and social isolation process was asked to them with an open-ended question. Descriptive analysis of the answers was carried out by two different researchers and the answers given to the question were categorised to form certain sub-themes. A total of 899 participants answered this question, 199 people (22·14 %) stated that they did not feel full during the social isolation process, their portion sizes increased, they continued to eat even though they were full or they continued to consume food between meals, and these answers were gathered under the main title ‘increased uncontrolled eating behaviour’. In total, 151 people (16·12 %) participating in the study declared that their consumption of certain types of foods, especially those with high carbohydrate content, increased. In this process that there would be a change of people’s eating behaviour, it is an expected result that individuals have been inclined to consume certain types of foods. In a study conducted by Van Strien and Koenders in 2014 with 553 female and 911 male participants, it was shown that there was a positive correlation between emotional eating, uncontrolled eating and restrictive eating behaviours and sugary/fatty food intake in both men and women^(27)^. This study supports the uncontrolled eating behaviours observed in individuals who participated in our research and their behaviour towards certain foods. It was questioned whether there was a change in the body weight of the individuals during the COVID-19 pandemic and social isolation process, and 35 % of the participants declared increased body weight in this process. Beyond triggering a chronic inflammatory state, nutritional behaviour changes that occur during this period may be associated with an increased risk of serious complications, obesity, heart disease, diabetes and lung disease due to COVID-19^(26)^. In a study conducted with forty-four women aged 25–42 years (2018) to examine the effects of emotions caused by suddenly changing life events on nutritional intake, negative emotional changes were shown to affect individuals’ body weight by causing higher energy intake than ordinary daily life^(28)^. Obesity, which is accepted as an important public health problem, affects 13 % of individuals over the age of 18 years worldwide and 32 % of individuals over the age of 20 years in our country^(29,30)^. It is estimated that obesity will affect 2·7 billion people worldwide. People who are overweight or obese are under high risk at having diseases such as CVD, cancer and type 2 diabetes mellitus. Since the prevalence of obesity is increasing rapidly, cautions and interventions that may help to decrease or prevent the harm should be taken immediately. It seems that obese people are more tempted to have uncontrolled eating behaviour then people who have normal body weight^(31)^. The results of this research show that uncontrolled eating behaviour increased significantly in individuals in the normal or overweight category according to BMI value. It should be kept in mind that failure to maintain the current status in individuals with normal BMI values or maintaining uncontrolled eating behaviour in overweight individuals who are at risk for the development of obesity may result in an increase in obesity prevalence in the long term.

The data of this research were collected through an online survey created on *Google Forms.* Online surveys are considered as one of the most effective and practical methods to evaluate and monitor information, attitudes and behaviours in infectious disease outbreaks with high spreading rate^(32)^. In this study, in which nutritional behaviour change was questioned, the use of the online survey method instead of face-to-face surveys reduced the risk of participants showing bias when answering questions. Thanks to this method, data were collected not only at the provincial level, but across the country, and the data collection process was completed more quickly. Another strength of the study is that, in addition to the quantitative data obtained from the questionnaire and scale, the qualitative data were also collected and evaluated. In this way, it has become possible to interpret the attitudes and behaviour changes of individuals about their diet in many ways. The most important limitation of using online questionnaire in our study is that individuals who are illiterate and/or do not have Internet access cannot be reached within the scope of this research. Other limitations of the study are that the TFEQ-R18 results and the body weight changes of the individuals were evaluated retrospectively, and the body weight values were based on the self-statements of the individuals. The reason why a different method could not be followed for this data is that people could only be reached online in this process of the social isolation. For future studies that will evaluate eating behaviour during social isolation, the mood state and stress level can also be examined for better understanding.

As a result, in this study, it was shown that there was a significant increase in emotional eating and uncontrolled eating behaviours of individuals in the pandemic and social isolation process. In this process, most of the individuals participating in the research declared that they have increased in body weight. The most obvious changes in their nutritional behaviour are increased uncontrolled eating behaviour, increased consumption of certain foods (usually high in carbohydrates) and altered diet. For this reason, a wide range of nutritional training mainly on adequate and balanced nutrition, rational use of food resources, food waste and food hygiene should be given to the society by dietitians during the pandemic and social isolation process, and afterwards. Establishing this awareness in the society in the early period and preventing nutritional errors will provide support for corrective actions to be taken regarding nutritional health problems that may occur in the future.

## References

[1] Wang D , Hu B , Hu C et al. (2020) Clinical characteristics of 138 hospitalized patients with 2019 novel coronavirus-infected pneumonia in Wuhan, China. JAMA 323, 1061–1069. 32031570 10.1001/jama.2020.1585 PMC7042881

[2] Zhou P , Yang XL , Wang XG et al. (2020) A pneumonia outbreak associated with a new coronavirus of probable bat origin. Nature 579, 270–273. 32015507 10.1038/s41586-020-2012-7 PMC7095418

[3] WHO (2020) Naming the coronavirus disease (COVID-19) and the virus that causes it. https://www.who.int/emergencies/diseases/novel-coronavirus-2019/technical-guidance/naming-the-coronavirus-disease-(covid-2019)-and-the-virus-that-causes-it (accessed April 2020).

[4] Meo SA , Alhowikan AM , Al-Khlaiwi T et al. (2020) Novel coronavirus 2019-nCoV: prevalence, biological and clinical characteristics comparison with SARS-CoV and MERS-CoV. Eur Rev Med Pharmacol Sci 24, 2012–2019. 32141570 10.26355/eurrev_202002_20379

[5] WHO (2020) Coronavirus disease (COVID-19) outbreak: WHO announces COVID-19 outbreak a pandemic. http://www.euro.who.int/en/health-topics/health-emergencies/coronavirus-covid-19/news/news/2020/3/who-announces-covid-19-outbreak-a-pandemic (accessed April 2020).

[6] Jebril N (2020) World Health Organization declared a pandemic public health menace: a systematic review of the coronavirus disease 2019 “covid-19”, up to 26th March 2020. https://www.psychosocial.com/article/PR290311/25748/ (accessed April 2020).

[7] Dietitians of Canada (2020) Advice for general public about COVID-19. https://www.dietitians.ca/News/2020/Advice-for-the-general-public-about-COVID-19 (accessed April 2020).

[8] Academy of Nutrition and Dietetics (2020) Concerns regarding COVID-19. https://www.eatright.org/coronavirus (accessed April 2020).

[9] Naja F & Hamadeh R (2020) Nutrition amid the COVID-19 pandemic: a multi-level framework for action. Eur J Clin Nutr 74, 1117–1121. 32313188 10.1038/s41430-020-0634-3 PMC7167535

[10] Caldwell AE & Sayer RD (2019) Evolutionary considerations on social status, eating behavior and obesity. Appetite 142, 238–248. 10.1016/j.appet.2018.07.028 PMC7039671 30078673

[11] Ohara K , Mase T , Kouda K et al. (2019) Association of anthropometric status, perceived stress, and personality traits with eating behavior in university students. Eat Weight Disord 24, 521–531. 30656613 10.1007/s40519-018-00637-w

[12] Verzijl CL , Ahlich E , Schlauch RC et al. (2018) The role of craving in emotional and uncontrolled eating. Appetite 123, 146–151. 29253669 10.1016/j.appet.2017.12.014 PMC5817024

[13] Deroost N & Cserjési R (2018) Attentional avoidance of emotional information in emotional eating. Psychiatry Res 269, 172–177. 30149274 10.1016/j.psychres.2018.08.053

[14] Booth C , Spronk D , Grol M et al. (2018) Uncontrolled eating in adolescents: the role of impulsivity and automatic approach bias for food. Appetite 120, 636–643. 29066344 10.1016/j.appet.2017.10.024 PMC5689136

[15] Cornelis MC , Rimm EB , Curhan GC et al. (2014) Obesity susceptibility loci and uncontrolled eating, emotional eating and cognitive restraint behaviors in men and women. Obesity 22, 135–141. 10.1002/oby.20592 PMC3858422 23929626

[16] Linardon J (2018) The relationship between dietary restraint and binge eating: examining eating-related self-efficacy as a moderator. Appetite 127, 126–129. 29727720 10.1016/j.appet.2018.04.026

[17] Kıraç D , Kapsar EÇ , Avcılar T et al. (2015) A new method in investigation of obesity-related eating behaviors ‘three-factor eating questionnaire’. MÜSBED 5, 162–169.

[18] Stunkard AJ & Messick S (1985) The three-factor eating questionnaire to measure dietary restraint, disinhibition and hunger. J Psychosom Res 29, 71–83. 3981480 10.1016/0022-3999(85)90010-8

[19] WHO (2020) Body mass index–BMI. https://www.euro.who.int/en/health-topics/disease-prevention/nutrition/a-healthy-lifestyle/body-mass-index-bmi (accessed August 2020).

[20] Hayran O & Özbek H (2017) Research and Statistical Methods in Health Sciences, 2nd ed İstanbul: Nobel Medicine Bookstore.

[21] Coffey A & Atkinson P (1996) Making Sense of Qualitative Data: Complementary Research Strategies. London, Thousand Oaks, CA and New Delhi: Sage Publications.

[22] Higgins JP , Altman DG , Gøtzsche PC et al. (2011) The Cochrane Collaboration’s tool for assessing risk of bias in randomised trials. BMJ 343, d5928. 22008217 10.1136/bmj.d5928 PMC3196245

[23] Mattioli AV , Ballerini Puviani M , Nasi M et al. (2020) COVID-19 pandemic: the effects of quarantine on cardiovascular risk. Eur J Clin Nutr 74, 852–855. 32371988 10.1038/s41430-020-0646-z PMC7199203

[24] Pearl RL (2020) Weight stigma and the “Quarantine-15”. Obesity 28, 1180–1181. 32324954 10.1002/oby.22850 PMC7264559

[25] Koball AM , Meers MR , Storfer-Isser A et al. (2012) Eating when bored: revision of the emotional eating scale with a focus on boredom. Health Psychol 31, 521–524. 22004466 10.1037/a0025893

[26] Muscogiuri G , Barrea L , Savastano S et al. (2020) Nutritional recommendations for Covid-19 quarantine. Eur J Clin Nutr 74, 850–851. 32286533 10.1038/s41430-020-0635-2 PMC7155155

[27] Van Strien T & Koenders PG (2014) Effects of emotional eating and short sleep duration on weight gain in female employees. J Occup Environ Med 56, 659–66. 24905422 10.1097/JOM.0000000000000172

[28] Aguiar-Bloemer AC & Diez-Garcia RW (2018)Influence of emotions evoked by life events on food choice. Eat Weight Disord 23, 45–53. 29285746 10.1007/s40519-017-0468-8

[29] WHO (2019) Obesity and overweight. https://www.who.int/news-room/fact-sheets/detail/obesity-and-overweight (accessed April 2020).

[30] Satman İ , Alagol F , Omer B et al.(2011) Diabetes, Hypertension, Obesity and Endocrine Diseases Prevalence Study of Turkey II. https://www.turkendocrin.org/files/fileD_156.pdf (accessed April 2020).

[31] Benbaibeche H , Bounihi A & Koceir EA (2020) Leptin kevel as a biomarker of uncontrolled eating in obesity and overweight. Ir J Med Sci 44, 438–446. 10.1007/s11845-020-02316-1 32681271

[32] Eysenbach G (2020) Use of rapid online surveys to assess people’s perceptions during infectious disease outbreaks: a cross-sectional survey on COVID-19. J Med Internet Res 22, e18790. 32240094 10.2196/18790 PMC7124956

